# Evaluation of right-ventricular function by two-dimensional echocardiography and two-dimensional speckle-tracking echocardiography in patients with successful RCA CTO recanalization

**DOI:** 10.1007/s00392-023-02259-4

**Published:** 2023-08-01

**Authors:** Recha Blessing, Ioannis Drosos, Michael Molitor, Thomas Münzel, Philip Wenzel, Tommaso Gori, Zisis Dimitriadis

**Affiliations:** 1 Department of Cardiology, University Medical Center Mainz, Johannes Gutenberg University, Langenbeckstr. 1, 55131 Mainz, Germany; 2 German Center for Cardiovascular Research (DZHK), Partner Site Rhine-Main, Mainz, Germany; 3 Division of Cardiology, Department of Medicine III, Center of Internal Medicine, University Hospital Frankfurt, Goethe University Frankfurt am Main, Theodor-Stern-Kai 7, 60590 Frankfurt am Main, Germany; 4 Center for Thrombosis and Hemostasis (CTH), Johannes Gutenberg University, Mainz, Germany

**Keywords:** Chronic total occlusion (CTO), Percutaneous coronary intervention (PCI), Coronary artery disease, Right ventricle (RV), Two-dimensional speckle-tracking echocardiography (2DESTE)

## Abstract

**Objectives:**

Chronic total occlusion (CTO) of the right coronary artery (RCA) is common in patients with coronary artery disease. Although revascularization techniques and success rates have improved significantly in recent years, there are still no studies investigating possible effects of successful recanalization of RCA CTO on the right-ventricular (RV) function. With this study, we aimed to evaluate RV function after recanalization of the RCA by two-dimensional transthoracic echocardiography (2DE) and additional two-dimensional speckle-tracking echocardiography (2DSTE).

**Methods and results:**

Our analysis included 102 patients undergoing successful RCA CTO recanalization at the University Medical Center of Mainz. All patients underwent 2DE and 2DSTE to assess RV function before PCI procedure and 6 months after successful revascularization. We found an altered RV function in our collective at baseline assessed by 2DSTE with a significant improvement at 6 month follow-up (baseline RV free wall strain: − 20.7 [− 6.3 to − 32.0] % vs. − 23.4 [− 8.3 to − 39.3] % at follow-up, *p* < 0.001 and baseline RV global strain − 15.9 [− 6.0 to − 25.7] % vs. − 17.9 [− 7.0 to − 29.5] % at follow-up, *p* < 0.001).

**Conclusion:**

RV function was altered in patients with RCA CTO and showed significant improvement after successful recanalization. We also noticed an improvement in patient-reported clinical symptoms. Our study suggests that CTO procedure is a beneficial treatment option in symptomatic patients with RCA CTO.

## Introduction

The complex anatomy and the volume and pressure dependency represent a challenge for accurate assessment of the right ventricle (RV) with the conventional cardiovascular imaging techniques. As a result, assessment of the RV was neglected for a long time and studies focused on the left ventricle [1]. Impaired RV function is associated with a worse outcome of patients with pulmonary hypertension, heart failure and coronary artery disease [2–8]. RV function not only has prognostic relevance for patients, but also has a decisive influence on clinical symptoms and exercise capacity [9, 10]. The RV is routinely assessed by two-dimensional echocardiography (2DE). The conventional functional parameters TAPSE (tricuspid annular plane systolic excursion), 2D RV-FAC (fractional area change), and TDI S’ (Doppler-derived tricuspid lateral annular systolic velocity) are recommended by the American Society of Echocardiography and the European Association of Cardiovascular Imaging to assess the systolic function of the RV [11, 12]. More recently, two-dimensional speckle-tracking echocardiography (2DESTE) is increasingly becoming the focus of interest as a reproducible, accessible, and accurate method to investigate RV function [13–15].

Coronary artery disease is one of the leading causes of death worldwide [16]. Register studies show that 15–25% of patients with coronary artery disease have a chronic total occlusion (CTO) of at least one coronary artery [17].

Several studies have investigated previously the effect of successful CTO percutaneous coronary intervention (PCI) on left-ventricular function, but data on the RV function are lacking [18, 19]. With this study, we aimed to investigate RV function in patients with RCA CTO assessed by two-dimensional echocardiography and two-dimensional speckle-tracking echocardiography and whether successful RCA CTO PCI affects the right-ventricular function.

## Methods

Between August 2018 and May 2022, 124 patients with RCA CTO underwent successful recanalization at our institution. All patients signed an informed consent for this prospectively conducted study. 2DE and 2DESTE were performed at baseline (before RCA CTO PCI) and at 6 month follow-up after a surveillance coronary angiography confirmed a good result without the need of revascularization within the treated vessel.

**Fig. 1:**
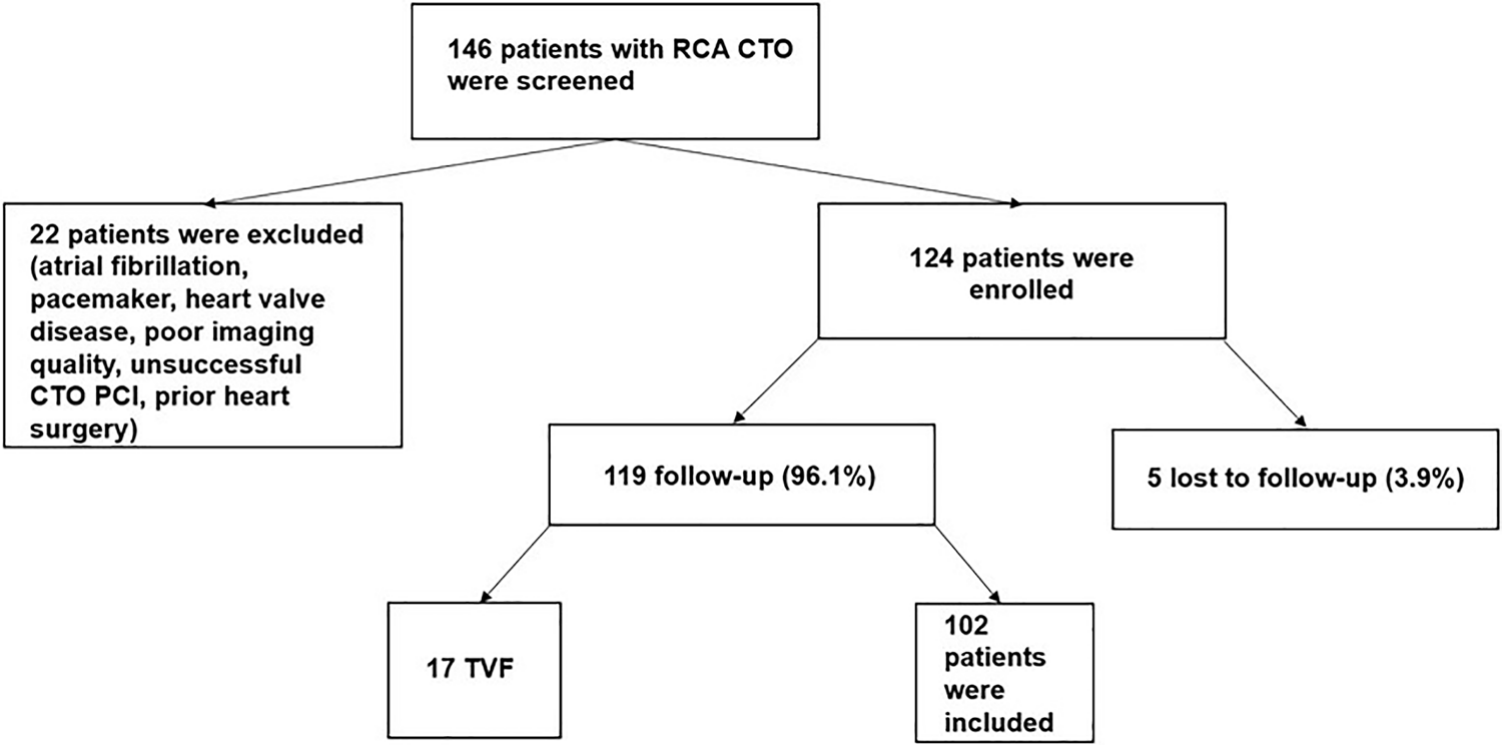
Flowchart of enrollment. TVF = target vessel failure (defined as presence of diameter restenosis > 50% by visual estimation, total re-occlusion, or any revascularization within the treated vessel at 6 month follow-up surveillance coronary angiography)

Patients with valve disease (moderate or severe valve regurgitation or stenosis), prior valve surgery or interventional repair, prior cardiac surgery, atrial fibrillation, paced rhythm, left or right bundle branch block, severe pulmonary, kidney (dialysis) or liver disease, pulmonary hypertension, cardiac surgery, or intervention in the period between study inclusion and follow-up were excluded. Inclusion criteria for our study were proof of viability, successful recanalization of RCA CTO with good angiographic result in the 6 months surveillance coronary angiography [absence of target vessel failure (TVF defined as presence of diameter restenosis > 50% by visual estimation, total re-occlusion, or any revascularization within the treated vessel at 6 months follow-up surveillance)], sinus rhythm, and sufficient 2-dimensional imaging quality. Only patients whose long-term medication had not changed by the time of the follow-up were included. 102 patients were included in the final analysis (Fig. 1). The local ethics committee approved the study protocol and registered it as a study in the DRKS.

### Standard echocardiography and two-dimensional strain echocardiography

**Fig. 2:**
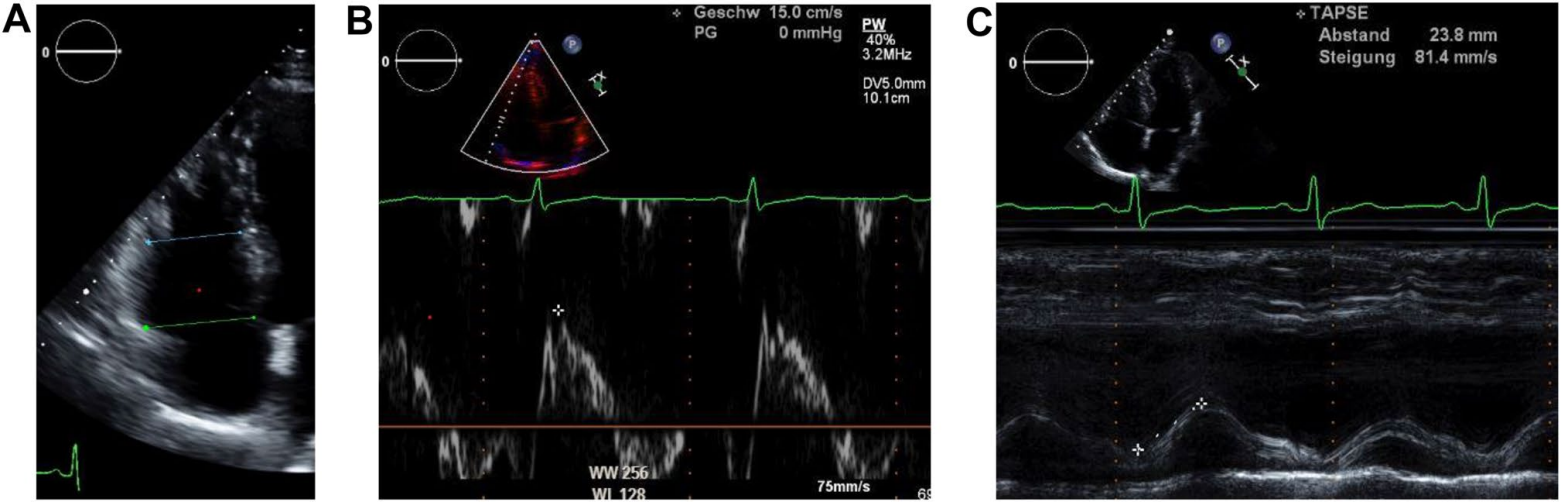
**A** RV diameter at base (green line): linear dimension measured end-diastolic at the basal one third of the RV; RV diameter mid-level (blue line): linear dimension measured end-diastolic in the middle third of the RV; **B** representative example of TAPSE (a parameter of RV longitudinal function and is measured from the tricuspid lateral annulus in M-Mode); **C** representative example of TDI S’ (the systolic velocity of lateral tricuspid annulus by pulsed tissue Doppler)

The measurements of the transthoracic echocardiography for quantification of the RV were selected based on the expert consensus document of the European Association of Cardiovascular Imaging. The right heart dimensions were evaluated using diameter at the base, diameter at the mid-level. Right ventricular function was assessed by TAPSE, 2D RV-FAC, and TDI S’ [20]. The assessment of these parameters was performed in accordance with the guidelines of the American Society of Echocardiography and the European Association of Cardiovascular Imaging [11, 12]. A representative example of these measurements is shown in Fig. 2A–C.

Conventional transthoracic echocardiography was performed at rest in a left lateral position using a Philips EPIQ 7 ultrasound system (Koninklijke Philips N.V., Amsterdam, The Netherlands). 2DE included two-dimensional views, including apical 4-chamber, apical 2-chamber, and parasternal long-axis views and Doppler imaging analysis. Two cardiac cycles were recorded and analyzed in each view.

**Fig. 3:**
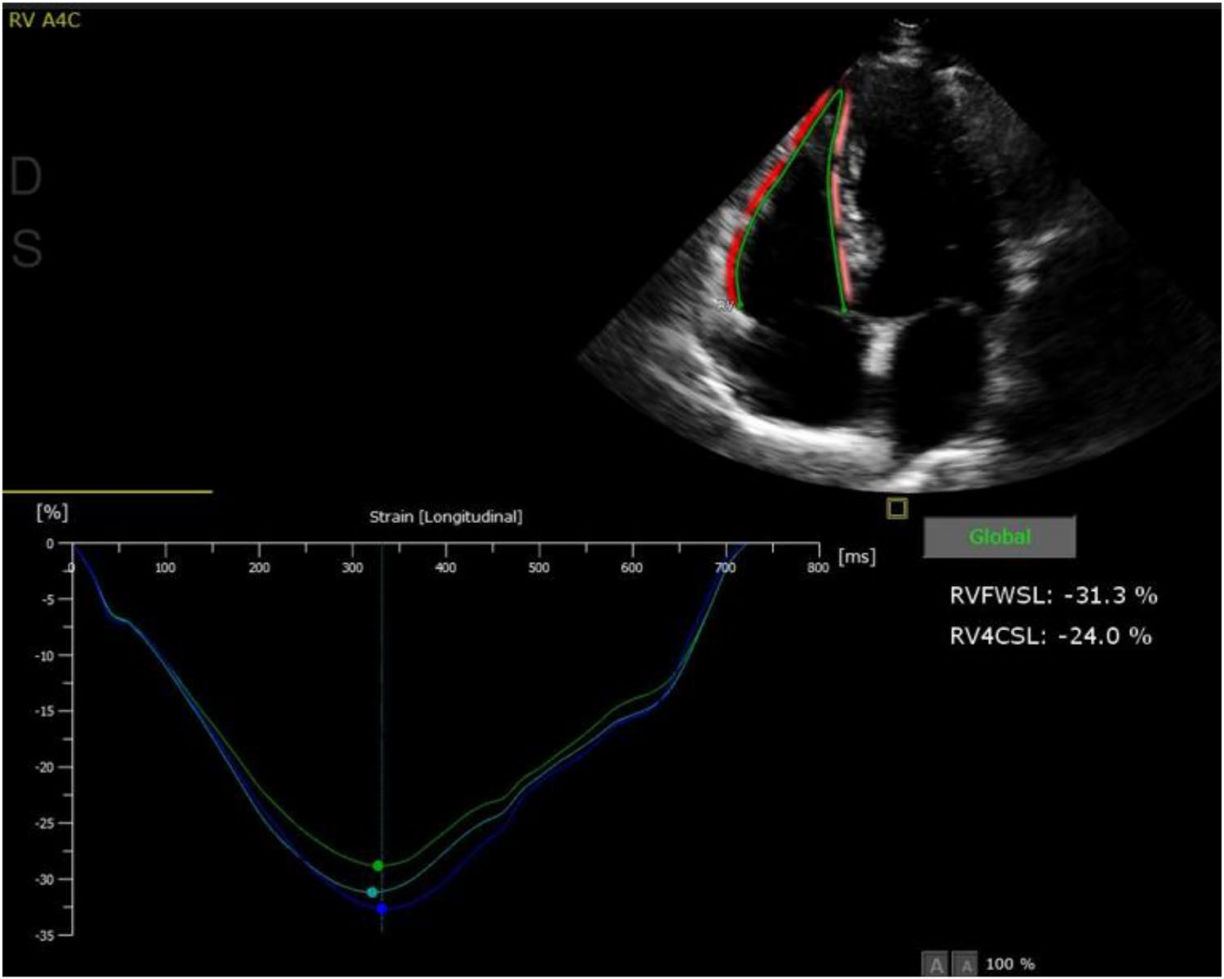
Representative example of RV strain imaging

The RV strain analysis [RV free wall systolic strain (%) and RV global (septal and free wall) systolic strain (%)] was performed offline using Q-LAB 13 (PHILIPS Andover, MA Koninklijke Philips Electronics N.V. 2019). Based on the recommendations of the consensus EACVI/ASE/Industry Task Force to standardize deformation imaging, the RV focused 4-chamber view was used, to visualize the entire RV and avoid shortening. RV's region of interest (ROI) was traced along the endocardial border as follows: tricuspid valve annulus, RV free wall, RV apex, septum, and ending at the opposite tricuspid annulus. The ROI was generated automatically, was checked, and, if necessary, the contours were adjusted manually (Fig. 3).

Angina was classified according to the Canadian Cardiovascular Society (CCS) classification and dyspnea based on NYHA (New York Heart Association).

### Statistical analysis

Normal distribution of the variables was tested by QQ-plot analysis, the Kolmogorov–Smirnov test, and Shapiro–Wilk test. Categorical data are presented as frequency and percentage, and normally distributed data as mean ± standard deviation and not normally distributed variables are presented as median and interquartile range. To investigate possible differences between baseline and follow-up values, we used the Wilcoxon signed-rank test. Correlations were tested with Spearman's Rho. To investigate possible effects of the right-ventricular side branch (RVSB) on the RV function, we formed four groups (RVSB occluded before and after PCI, RVSB not occluded before and after PCI, RVSB occluded before and not occluded after PCI, and RVSB not occluded before and occluded after PCI) and investigated possible differences between the groups with the Kruskal–Wallis test. A two-sided *p* value of < 0.05 was statistically significant. The statistical analysis was performed using SPSS (Version 23, IBM SPSS Statistics).

## Results

### Demographic and clinical baseline characteristics

Of the 102 patients in our final analysis, 72.5% were male with a median age of 66 years (44–80). The mean follow-up period was 187 ± 12.65 days. Median J-CTO Score was 2 (1–3) and most of the patients (54.9%) had Rentrop grade 2 of coronary collateral circulation. Baseline demographical and CTO characteristics are listed in Table 1.

**Table 1:** Baseline characteristics

	All patients (*n* = 102)
Demographics characteristics	
Age (yrs)	66 (44–80)
Male	74 (72.5)
BMI (kg/m^2^)	26.2 (20–42.4)
Diabetes mellitus	25 (24.5)
Hypertension	92 (90.2)
Hyperlipidemia	90 (88.2)
Smoker	36 (35.3)
Multivessel CAD	90 (88.2)
GFR (ml/min)	81.5 (9–117)
PAD	10 (9.8)
Previous MI	26 (25.5)
Previous PCI	71 (69.6)
CTO characteristics	
Balanced coronary circulation	71 (69.6)
Left dominant coronary circulation	29 (28.4)
Right dominant coronary circulation	2 (2.0)
J-CTO	2 (1–3)
Rentrop classification	
1	34 (33.3)
2	56 (54.9)
3	12 (11.8)
Werner classification	
1	57 (55.9)
2	35 (34.3)
3	10 (9.8)

Values are represented as *n* (%), median (minimum–maximum), or mean ± SD

*yrs* years, *BMI* body mass index, *CAD* coronary artery disease, *GFR* glomerular filtration rate, *PAD* peripheral artery disease, *MI* myocardial infarction, *PCI* percutaneous coronary intervention, *RVSD* dominant right-ventricular side branch

Clinical parameters at baseline are shown in Table 2. We found no difference when comparing BNP values at baseline and follow-up in our collective (*p* = 0.150). 65 of the enrolled patients reported an improvement in CCS class (63.7% and *p* < 0.001) and 51 reported an improvement in NYHA class (51.0% and *p* < 0.001). Complete freedom of angina was achieved in 72.5% of the patients in our collective and 58.8% of the patients reported to having no limitation of physical activity in daily life (NYHA stage 1) at follow-up.

**Table 2:** Clinical parameters baseline and follow-up

	Baseline (*n* = 102)	Follow-up (*n* = 102)	*p* value
NYHA			< 0.001
1	13 (12.7%)	60 (58.8%)	
2	69 (67.6%)	35 (34.8%)	
3	19 (18.6%)	6 (5.9%)	
4	1 (1.0%)	1 (1.0%)	
CCS			< 0.001
0	12 (11.8)	74 (72.5%)	
1	27 (26.5)	7 (6.9%)	
2	48 (47.1)	18 (17.6%)	
3	15 (14.7)	3 (2.9%)	
BNP	70 (10–788)	61.5 (10–892)	0.150
VCI	1.9 (1.7–2.7)	1.9 (1.6–2.9)	0.223

Values are represented as *n* (%) and median (minimum–maximum)

*p* value comparison of baseline and follow-up values

*NYHA* New York Heart Association stages, *CCS* Canadian Cardiovascular Society Classification, *BNP* brain natriuretic peptide pg/ml, *VCI* vena cava inferior (cm)

### Echocardiographic parameters

The measured RV diameters were within the normal ranges compared to the reference values recommended by the American Society of Echocardiography and the European Association of Cardiovascular Imaging. Median RV basal diameter was 36 mm (30–46) at baseline (reference values 33 ± 4 mm without significant change in the follow-up) (*p* 0.203), RV mid-level diameter was 30 mm (22–36) (reference value 27 ± 4 mm, *p* 0.374), and RV longitudinal diameter was 70 mm (56–86) (reference value 71 ± 6 mm, *p* 0.348). The analysis of the functional parameters at baseline was also within the normal ranges: TAPSE was 20 mm (reference value TAPSE > 17 mm), TDI S’ was 12.3 cm/s (reference value 9.5 cm/s) and FAC was 35.01% (reference values > 35%) in our collective. In our collective, 19 of 102 patients had an impaired TAPSE, and we were able to determine an improvement in all 19 patients in the 6-month follow-up (*p* < 0.001). 73 out of 102 patients showed an RV-FAC lower than 35%, we observed an improvement in 59 patients after a successful RCA CTO recanalization at the follow-up echocardiography (*p* < 0.001). 14 out of 102 patients had an impaired TDI and an improvement could be observed in all patients (*p* < 0.001).

We found significant increase of the functional parameter at follow-up, as shown in Table 3 [12]. Left-ventricular function assessed by left-ventricular ejection fraction (LVEF) and global longitudinal strain (GLS) showed no improvement after successful CTO PCI. In our collective, 77 (75.5%) patients had inferior or posterolateral hypokinesia prior to the recanalization. At follow-up, inferior or posterolateral hypokinesia was found in 66 (64.7%) of the patients (*p* < 0.001).

**Table 3:** Echocardiographic assessment of dimension and function

	Baseline (*n* = 102)	Follow-up (*n* = 102)	*p* value
Ventricular function			
RV basal diameter (mm)	36 (30–46)	36 (30–46)	0.203
RV mid diameter (mm)	30 (22–36)	30 (23–41)	0.374
RV longitudinal diameter (mm)	70 (56–86)	70 (57–88)	0.348
RV wall thickness (mm)	5 (3–8)	5 (3–8)	0.185
TDI S’ (cm/s)	12.30 (5.7–16.6)	12.75 (5.9–17.9)	< 0.001
TAPSE (mm)	20 (6.1–28)	22 (7.2–31)	< 0.001
FAC (%)	35.01 (11.21–60.51)	36.14 (17.54–64.06)	0.002
GLS (%)	− 15.65 ± 4.39	− 16.05 ± 4.13	0.179
LVEF (%)	55 (20–60)	55 (20–60)	0.179

Values are represented as mean ± SD and median (minimum–maximum)

*RV* right ventricle, *TAPSE* tricuspid anular plane systolic excursion, *FAC* fractional area change, *GLS* global longitudinal strain of left ventricle, *LVEF* left-ventricular ejection fraction, *mm* millimeter, cm centimeters, s seconds

**Fig. 4:**
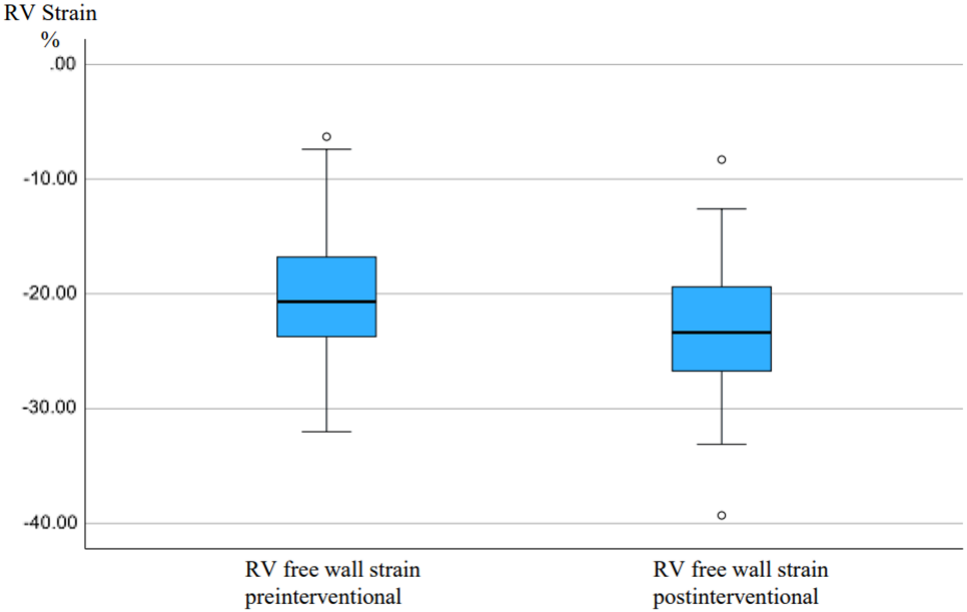
Boxplot diagram with RV free wall strain at baseline before RCA CTO PCI and 6 months after successful RCA CTO PCI

**Fig. 5:**
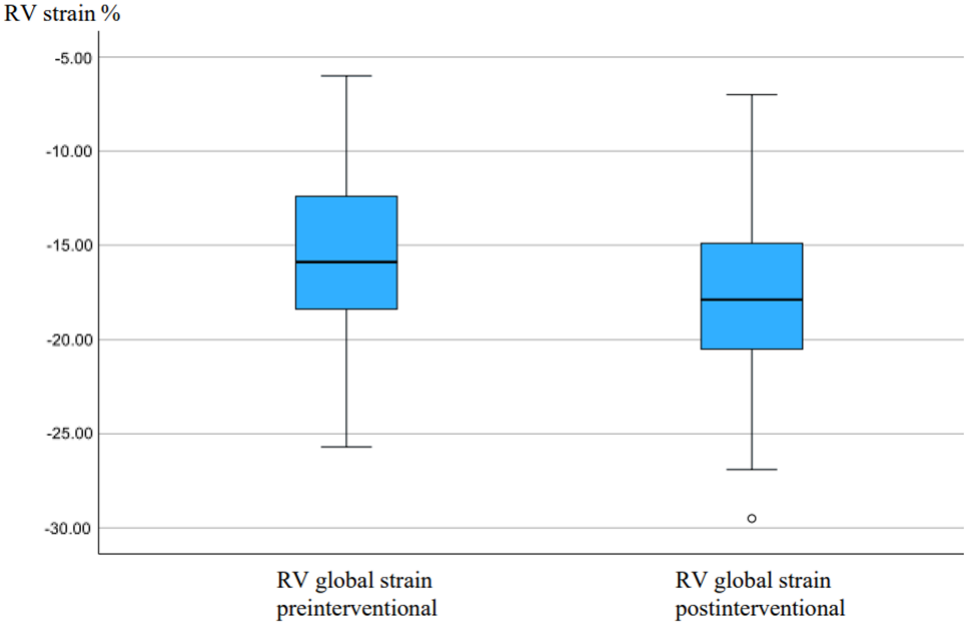
Boxplot diagram with RV global strain at baseline before RCA CTO PCI and 6 months after successful RCA CTO PCI

RV free wall strain was − 20.7 [− 6.3 to − 32.0]% at baseline and RV global strain was − 15.9 [− 6.0 to − 25.7]%, reference values being − 28.5 ± 4.8% for RV free wall strain, and − 24.5 ± 3.8% for RV global strain in healthy subjects [21]. The right-ventricular function of patients with RCA CTO was found to be altered assessed by speckle tracking in our analysis. We found a significant increase in both values at follow-up. The results of the RV function are presented in Table 4 and Figs. 4 and 5 (boxplot diagrams).

**Table 4:** RV function

	Baseline (*n* = 102)	Follow-up (*n* = 102)	*p* value
RV function			
RV free wall strain (%)	− 20.7 [− 6.3 to − 32.0]%	− 23.4 [− 8.3 to 39.3]%	< 0.001
RV global strain (%)	− 15.9 [− 6.0 to − 25.7]%	− 17.9 [− 7.0 to 29.5]%	< 0.001

Values are represented as mean and standard deviation

*RV* right ventricle

### Coronary collateral circulation and right-ventricular side branch

Correlation of coronary collateral circulation (Rentrop und Werner classification) with right-ventricular function after successful CTO PCI was calculated using Spearman’s Rho. We found no correlation between Werner’s classification (CC grade) and right-ventricular function (RV free wall strain und CC grade: correlation coefficient − 0.102 and *p* 0.307; RV global strain and CC grade: correlation coefficient 0.011 and *p* 0.912; RV free wall strain and Rentrop classification: correlation coefficient − 0.103 and *p* 0.301; RV global strain and Rentrop classification: correlation coefficient 0.012 and *p* 0.906).

We investigated whether the location of the CTO [proximal vs. distal to the dominant right-ventricular side branch (RVSB)] influenced right-ventricular function and found no difference between the groups (RV free wall *p* 0.527 and RV global strain *p* 0.325).

Patients were divided into the following groups: RVSB occluded before and after PCI, RVSB not occluded before and after PCI, RVSB occluded before and not occluded after PCI, and RVSB not occluded before and occluded after PCI. We found no difference between the groups the patients benefited regardless of side branch anatomy. Effects of the dominant RVSB on the result were tested with the Kruskal–Wallis test. The results are listed in Table 5.

**Table 5:** Effect of dominant right-ventricle side branch

	*n* = 102	*p* value
RVSB occluded before and after PCI	7	
RVSB not occluded before and after PCI	87	
RVSB occluded before and not occluded after PCI	5	
RVSB not occluded before and occluded after PCI	3	
RV global strain		0.937
RV free wall strain		0.300

Values are represented as *n* (%)

*RVSB* right-ventricle side branch, *RV* right ventricle, *PCI* percutaneous coronary intervention

### Reproducibility

Inter-observer reproducibility was tested at baseline and follow-up values for RV free wall and global strain. Inter-observer reproducibility was good (intraclass correlation coefficient for RV free wall strain before PCI: 0.84, 95% CI 0.696–0.904, RV free wall strain after PCI: 0.825, 95% CI 0.683–0.902, RV global strain before PCI: 0.849, 95% CI 0.707–0.918, and RV global strain after PCI: 0.797, 95% CI 0.644–0.884) as determined by intraclass correlation coefficients.

## Discussion

To the best of our knowledge, this is the first study analyzing RV function in patients with CTO of the RCA. We assessed RV function with longitudinal strain at baseline and 6 months after successful CTO PCI. Compared to other studies, we only included patients with a good angiographic result in the 6 month surveillance coronary angiography to ensure that the effect on RV function is mainly based on CTO PCI. The main findings of the study were: (1) right-ventricular function is altered in patients with RCA CTO, (2) right-ventricular function improved significantly after successful CTO PCI, and (3) the effect on right-ventricular function is independent of coronary collateralization circulation and the anatomy of the dominant right-ventricular side branch.

RV function is an essential parameter for the prognosis and treatment of patients with various diseases, especially after cardiac surgery or interventional therapies [2–4, 14]. On top of this, it is a relevant predictor for the functional status and exercise capacity of patients which underlines the importance of an accurate assessment of the RV [1]. The linear diameters and conventional parameters of RV function (FAC, TAPSE, and TDI S’) are recommended as part of clinical routine [20]. We found the quantitative parameters and the conventional measurement of the RV function to be within reference values in our collective. However, the assessment of the RV with the conventional parameters has some limitations due to its complex anatomy [thin and highly trabeculated RV wall, dependency of the RV function on preload and afterload, angle dependency of the examination, and the contraction pattern of the RV (longitudinal contraction accounts for 75% of RV contraction)] [1, 14, 22, 23]. Following these limitations, longitudinal strain analysis by 2D speckle tracking should be performed for accurate imaging of the RV. Longitudinal strain analysis already detects subclinical impairment and subtle changes in RV function and is less afterload dependent compared to the conventional parameters [14, 20, 22]. Park et al. demonstrated a good correlation of the right-ventricular longitudinal strain with invasive parameters (cardiac index and pulmonary vascular resistance) and functional parameters (BNP and 6-min walking test) [24]. Based on this scientific knowledge, longitudinal strain of the right ventricle is also recommended in pressure or volume overload conditions and provides valid assessment [22, 25]. To investigate the role of RCA CTO PCI on RV function precisely and minimize the effect of potential influencing factors (e.g., volume and pressure overload), we performed strain analysis and excluded patients with pulmonary hypertension and severe lung or valvular disease. Additionally, we monitored brain natriuretic peptide values and the diameter of the inferior vena cava at baseline and follow-up. We found no significant difference in these parameters.

The benefits of CTO PCI are still controversial. There is evidence that patients benefit from CTO PCI with improved clinical symptoms and quality of life [26–29]. In contrast, study results of the prognostic benefit and the effect on the left-ventricular function are still inconsistent [18, 19, 27, 29, 30]. One reason for the controversial results could be the different study conditions (examinations at rest vs. stress), as studies have especially revealed exercise-induced ischemia of the myocardium of the CTO territory. The REVASC Trial investigated the effect of CTO PCI on the left-ventricular function. The segmental wall thickening of the CTO territory was assessed by cardiac magnetic resonance imaging (CMR) at rest at baseline and 6 month follow-up. In about 60% of the patients, the CTO was localized in the RCA. The authors reported no improvement of the segmental wall thickening and other left-ventricular indices after 6 months [19]. In contrast to this study, Bucciarelli-Ducci et al. showed a significant improvement of LEVF in patients after successful CTO PCI assessed by stress perfusion imaging CMR [31]. The IMPACTOR-CTO trial focused on patients with RCA CTO to study the effects of CTO PCI vs. optimal medical therapy (OMT) on inducible myocardial ischemia burden in this special collective. Adenosine stress cardiac magnetic resonance, 6-min walk test, and Short Form-36 Health Survey were performed at baseline, after 2 and 12 months. Endpoints were defined as: decrease in inducible myocardial ischemia burden, changes in 6-min walk test distance, and quality of life. In the follow-up, inducible myocardial ischemia burden was lower and decreased significantly in the CTO PCI collective, 6-min walking distance significantly increased in the CTO PCI collective, and the scales of the Short Form-36 Health Survey improved compared to the OMT collective. These effects could not be shown in the OMT collective and no significance was reached in the OMT collective for the endpoints. The decrease of inducible myocardial ischemia burden indicates an improved perfusion of the CTO supplied territory after PCI [26]. These results underline the importance of studying the ventricular function not only at rest but also under stress conditions. Our analysis is in good agreement with the findings above. The comparison of the left-ventricular indices showed no difference between baseline and follow-up. The mean GLS was within the borderline range (16–18%) in our collective without improvement at follow-up [20, 32]. We expected these results as Pereztol-Valdes et al. already showed that none of the 17 segments of the LV is exclusively perfused by the RCA [33, 34]. Since the right ventricle is mainly supplied with blood by the RCA, we hypothesized that the improved perfusion of the CTO supplied territory after CTO PCI has a positive effect on the RV function. With our analysis, we were able to confirm this hypothesis using global and free wall strain assessment. In addition to improved perfusion, other reasons for our findings could be the improved microvascular function and the increased trainings capacity of our patients due to reduction of clinical symptoms (especially angina relief). These findings support the benefits of CTO PCI in patients with RCA CTO and clinical symptoms.

## Conclusion

Our single-center experience demonstrates a significant improvement of the right-ventricular function assessed by two-dimensional echocardiography and longitudinal strain imaging in patients with RCA CTO after successful recanalization. We also found an improvement in patient-reported exercise tolerance in daily life (NYHA classification) and angina symptoms (CCS classification).

### Limitations

This study has a single-center prospective design with a limited sample size and lacks of a control group. Our results, and their potential clinical impact, should be tested in a larger cohort. The longitudinal strain analysis is recommended for an accurate assessment of the right-ventricular function, but the results are dependent on the ankle and pre- and afterload conditions. We tried to reduce the influence of these factors by patients’ selection (exclusion criteria, e.g., pulmonary hypertension) and use of clinical parameters (BNP, VCI) to assess hemodynamic conditions. 2 DE was performed in patients with normotension, sinus rhythm, and no lung disease. However, the RV function is influenced by several factors, which should be taken into account when interpreting our data. Due to the size of our collective and the follow-up period of 6 months, we are not able to evaluate prognostic implications of RCA CTO PCI.

## Data Availability

The dataset generated and/or analyzed during the current study are available from the corresponding author on reasonable request.

## Abbreviations

CTO: Chronic total occlusion
BNP: Brain natriuretic peptide
CAD: Coronary artery disease
CCS: Canadian Cardiovascular Society Classification
FAC: Fractional area change
GFR: Glomerular filtration rate
GLS: Global longitudinal strain
LVEF: Left-ventricular ejection fraction
MI: Myocardial infarction
NYHA: New York Heart Association
OMT: Optimal medical therapy
PAD: Peripheral artery disease
PCI: Percutaneous coronary intervention
RCA: Right coronary artery
ROI: Region of interest
RV: Right ventricle
RVSB: Right-ventricular side branch
TVF: Target vessel failure
VCI: Vena cava inferior
2DE: Two-dimensional transthoracic echocardiography
2DSTE: Two-dimensional speckle-tracking echocardiography
